# Advances in the Interpretation of Frequency-Dependent Nuclear Magnetic Resonance Measurements from Porous Material

**DOI:** 10.3390/molecules24203688

**Published:** 2019-10-14

**Authors:** David Faux, Rémi Kogon, Villiam Bortolotti, Peter McDonald

**Affiliations:** 1 Department of Physics, University of Surrey, Guildford, Surrey GU2 7XH, UK; p.mcdonald@surrey.ac.uk; 2 Department of Physics and Astronomy, Viale Berti Pichat 6/2, 40127 Bologna, Italy; remialbert.kogon2@unibo.it; 3 Department of Civil, Chemical, Environmental, and Materials Engineering, Via Terracini 28, 40126 Bologna, Italy; villiam.bortolotti@unibo.it

**Keywords:** fast-field cycling, nuclear magnetic resonance, relaxation rate, porous material, diffusion

## Abstract

Fast-field-cycling nuclear magnetic resonance (FFC-NMR) is a powerful technique for non-destructively probing the properties of fluids contained within the pores of porous materials. FFC-NMR measures the spin–lattice relaxation rate $R_{1}\left(f\right)$ as a function of NMR frequency *f* over the kHz to MHz range. The shape and magnitude of the $R_{1}\left(f\right)$ dispersion curve is exquisitely sensitive to the relative motion of pairs of spins over time scales of picoseconds to microseconds. To extract information on the nano-scale dynamics of spins, it is necessary to identify a model that describes the relative motion of pairs of spins, to translate the model dynamics to a prediction of $R_{1}\left(f\right)$ and then to fit to the experimental dispersion. The principles underpinning one such model, the 3τ model, are described here. We present a new fitting package using the 3τ model, called 3TM, that allows users to achieve excellent fits to experimental relaxation rates over the full frequency range to yield five material properties and much additional derived information. 3TM is demonstrated on historic data for mortar and plaster paste samples.

## 1. Introduction

This article is concerned with the application of fast-field-cycling nuclear magnetic resonance (FFC-NMR) to porous materials. Proton NMR is the most effective laboratory-based technique for non-destructively probing the dynamics of proton spins in porous material, and NMR relaxometry accesses the nano-scale dynamics. Measurements yield the nuclear spin–lattice (longitudinal) relaxation rate, $R_{1}$, of ${}^{1}$H nuclei [1,2]. In an FFC-NMR experiment, the proton resonant frequency is altered by rapidly changing the strength of the applied magnetic field, allowing $R_{1}$ to be measured as a function of Larmor angular frequency ω=2πf for frequencies *f* typically from 10 kHz to 40 MHz. The broad frequency range accessible in an FFC-NMR experiment captures information on the dynamical processes of fluid spins over time scales of picoseconds to microseconds [2].

Fast-field-cycling NMR has been applied to a vast range of porous materials. Any system that contains proton-bearing fluid is accessible to FFC-NMR. Examples include rock, shales and mudstones which contain hydrocarbon and water mixtures [3,4,5,6,7,8], cement-based material including mortar, plaster and dental resins [5,9,10,11,12,13,14,15,16,17,18,19], zeolites [20] for water desalination, purification and catalysis, clays [3,20,21], wood [22], silicates and glasses [5,23,24,25,26,27], plus foodstuffs and a host of polymeric, protein and other biological materials.

The frequency-dependent relaxation rate $R_{1}\left(f\right)$ arises due to fluctuations in magnetic dipole–dipole interactions between pairs of spins in relative motion. The fluid contained in the porous structure of the material can exist in a wide range of environments due to the complexity of the internal structure of porous materials. Water molecules at a surface will be orientated and rotate, vibrate and move in a manner that is dependent on the surface morphology and the local fluid environment [28]. Surface dynamics will be influenced by defects, local atomic restructuring, steps and surface termination. Water molecules in the pore bulk may be bound to impurity ions and there may be proton spin diffusion in the absence of the physical movement of molecules. All these effects, and others, contribute to the measured relaxation rates.

Porous materials can be divided into two classes for the purpose of relaxation analysis: those in which paramagnetic ions are present, and those in which paramagnetic ions are absent. If paramagnetic ions are present, the relaxation rate is dominated by the dipolar interaction due to the relative motion of proton spins with respect to the (fixed or mobile) paramagnetic ions. Relaxation due to proton–proton interactions is normally negligible by comparison. The paramagnetic ions may be fixed because they are contained within the solid material, for example as impurities, or in motion because they exist as aqueous ions. The study of NMR relaxation from solutions containing paramagnetic species is an important area of research due to their use as contrast agents for magnetic resonance imaging and in biochemistry [29]. The model presented in this article neglects relaxation associated with aqueous paramagnetic ions.

Relaxation in cementitious material, clays and rock is normally dominated by the presence of paramagnetic impurity ions contained in the solid. Porous materials in which paramagnetic ions are absent may include limestones, plaster, synthetic clays, organo-metallics, some silicates, zeolites and glasses. Here, the frequency-dependent relaxation rate $R_{1}\left(f\right)$ is dominated by the combinations of interactions between pairs of ${}^{1}$H spins in relative motion.

**Figure 1:**
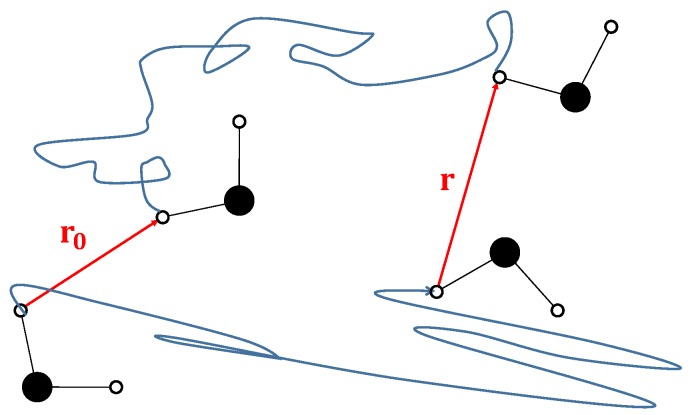
The figure shows, at left, two water molecules at time t=0. A pair of intermolecular hydrogen spins are connected by a vector $r_{0}$. The molecules diffuse within the porous material and the same two hydrogen atoms are separated by vector r after a time *t*. The frequency-dependent relaxation rate $R_{1}\left(f\right)$ is determined by the probability density function describing the probability that a pair of spins are separated by $r_{0}$ at t=0 and by r at time *t*.

The $R_{1}\left(f\right)$ dispersion curve obtained from an FFC-NMR experiment captures information on the relative motion of pairs of spins over time scales of many orders of magnitude. The challenge lies in interpreting the dispersion curves. A dynamical model must describe the relative motion of fluid in the bulk and at the surfaces of pores of varied geometry and connectivity. Consider Figure 1, which shows two water molecules at time t=0 with a pair of inter-molecular ${}^{1}$H spins separated by a vector $r_{0}$. The two water molecules diffuse, and at a time *t* later the vector connecting the two ${}^{1}$H spins becomes r, as shown in Figure 1. The dynamics are captured by the probability density function $P(r_{0}\cap r,t)$, which describes the probability that a pair of spins are separated by $r_{0}$ at t=0 and by r at time *t*. The strategy is to devise a model that captures the key physics of the fluid dynamics through $P(r_{0}\cap r,t)$ dependent on a set of parameters, to transform the model to a theoretical prediction of $R_{1}\left(f\right)$ and, finally, to fit to the experimental FFC-NMR data.

This article summarises recent advances in the 3τ model for the prediction of $R_{1}\left(f\right)$ [30,31,32]. The model builds upon the work of others who previously devised simplified models that captured some of the key physics but were limited in the number of parameters that could be reliably determined. The principles underpinning the 3τ model are described, and the innovations that have allowed the model to extract five characterising material properties from dispersion data are explained in Section 2. The application of the model is illustrated in Section 3 through two historic datasets on a hydrated mortar and a plaster paste.

## 2. The 3τ Model

In this section, the physical principles of the 3τ model which allows the calculation of the spin-pair probability density function $P(r,t\cap r_{0})$ are described, and the simplifications, approximations and computational processes by which $P(r,t\cap r_{0})$ is translated into a prediction of the relaxation rate $R_{1}\left(f\right)$ are explained.

### 2.1. The Physics behind the 3τ Model

**Figure 2:**
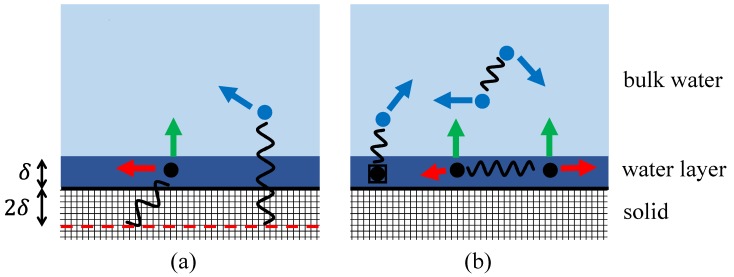
The arrows represent in-layer spin diffusion (red), desorption (green) and bulk diffusion (blue). In (**a**), a measurement of the relaxation rate $R_{1}\left(f\right)$ is dominated by the interaction of fixed paramagnetic impurities with both the water in the surface layer and in bulk. The density of paramagnetic impurities is modelled by a single effective layer (dashed line) placed 2δ below the surface. In (**b**), $R_{1}\left(f\right)$ is due to layer–layer, bulk–bulk and bulk–layer interactions. The surface layer of water is assumed to be δ=0.27 nm thick.

The physics underpinning the 3τ model is presented in Figure 2, which shows a fluid (assumed here to be water for convenience) at a flat pore surface. The water may occupy one of two distinct environments: surface or bulk. The water at the pore surface moves slowly parallel to the surface with a characteristic diffusion time constant labelled $\tau_{\ell }$. $\tau_{\ell }$ is related to the surface water diffusion coefficient by $D_{\ell }=\delta^{2}/6\tau_{\ell }$, where δ=0.27 nm is a standard convenient nano-scale distance approximately equivalent to the inter-molecular distance between hydrogen atoms in water, or the approximate thickness of a “layer” of water. Indeed, the thickness of the surface layer is fixed in the model as δ. The remaining water is labelled as “bulk” water, and it too has a characteristic diffusion time constant defined by the bulk water diffusion coefficient $D_{b}=\delta^{2}/6\tau_{b}$. The value of $\tau_{b}$ is about 5.3 ps for pure water at room temperature, which means that a water molecule takes on average 5.3 ps to move 0.27 nm. The third time constant is $\tau_{d}$, which is the characteristic time a water molecule remains at the surface before desorption under the assumption that surface spins desorb as $\exp(-t/\tau_{d})$. Typically, $\tau_{d}\approx \tau_{\ell }$. The physical principles of the 3τ model are grounded in molecular dynamics simulations [33,34,35] which confirm that the important water environments and that the key dynamical motions have been identified. We note that, in principle, numerical simulation techniques such as molecular dynamics [36] and random walk methods [37] may be used to compute the dipolar correlation functions, but that neither approach is suitable for executing fits to experimental data.

Porous materials may be divided into two classes: those in which the FFC-NMR relaxation rate is dominated by the interaction of water with the electronic spins associated with paramagnetic impurities in the solid, and those systems which do not contain paramagnetic relaxation centres. The two systems are illustrated in Figure 2. In (a), the relaxation is dominated by the presence of fixed paramagnetic impurities contained in the solid material of the pores. The paramagnetic impurities are of course distributed throughout the solid material, not necessarily uniformly. At high concentrations, the impurities tend to cluster. Building on earlier work [3,4,5,6,7], the 3τ model reduces the complex distribution of paramagnetic impurities to a single effective layer with a characteristic paramagnetic spin density $N_{\sigma }$. This is represented by the dashed line in Figure 2a. The interaction between the paramagnetic impurities and the water is a very strong function of the distance, and so the effective paramagnetic layer would be expected to be placed close to the surface. In the 3τ model, the paramagnetic layer is placed at a distance 2δ from the surface.

In the second class of porous systems (i.e., those without paramagnetic centres), relaxation takes place due to the relative motion of pairs of ${}^{1}$H spins. This is illustrated in Figure 2b, which identifies the surface water and the bulk water as the two water environments and illustrates the relevant spin–spin interactions. The bulk water spin density is $N_{b}=$ 66.6 spins/nm${}^{3}$ and the surface water density is normally assumed to be the same. The relaxation rate dispersion $R_{1}\left(f\right)$ takes contributions from pairs of spins, both of which are contained within the surface layer at time t=0 and at time *t*, a bulk–bulk spin contribution and a layer–bulk contribution. In the latter, because the surface water moves so slowly compared to the bulk, the layer water can be assumed to be fixed.

### 2.2. Calculating the Spin–Lattice Relaxation Rate Dispersion $R_{1}\left(f\right)$

Earlier, it was stated that the dynamics of spin pairs are captured in the spin-pair probability density function $P(r,t\cap r_{0})$. The task of a model is to determine the spin-pair probability density function for the specific interaction to be considered. This is normally obtained as a solution to the diffusion equation for the specific geometry of the problem. The second step is to determine the time-dependent dipolar correlation function G(t). A separate function G(t) is determined for each of the interactions illustrated in Figure 2.

The dipolar correlation function G(t) is obtained from the expression

(1) $$\begin{array}{c} G\left(t\right)=\frac{4\pi }{5}\int_{R^{3}}\int_{R_{0}^{3}}\left[\;\sum_{M=-2}^{2}\frac{Y_{2M}\left(x_{0},y_{0},z_{0}\right)\;Y_{2M}^{*}\left(x,y,z\right)}{r_{0}^{3}\;r^{3}}\right]P\left(r,t\cap r_{0}\right)\;d^{3}r_{0}\;\;d^{3}r. \end{array}$$

Equation (1) incorporates powder-averaging assuming a uniform distribution of randomly-orientated pores reflecting the usual experimental practice of using powdered samples [33] or accepting that un-powdered samples comprise large numbers of pores randomly orientated within. The *Y* are the spherical harmonic functions of degree 2 (where the asterisk superscript represents the complex conjugate) expressed here in terms of the Cartesian coordinates of the spin-pair vectors but cylindrical coordinates or spherical polar coordinates would be used depending on the geometry of the fluid volume. The calculation expressed by Equation (1) must be undertaken for each of the interactions indicated in Figure 2. It is quite clear that this is a challenging task, as the integrations are not necessarily analytic. Therefore, numerical computations are necessary if representative correlation functions are to be obtained. For the 3τ model, the details of these calculations were presented in back-to-back articles in 2017 for assumed planar pores [30,31].

The third step Fourier transforms G(t) to obtain the spectral density function J(ω), and may be written

(2) $$\begin{array}{ccc} J(\omega ) & = & 2\int_{0}^{\infty }\;\;G\left(t\right)\;\cos\omega t\;dt. \end{array}$$

Since G(t) is obtained via a numerical computation, it follows that a numerical integration must be undertaken to generate the spectral density functions J(ω). However, this is also far from straightforward. To obtain a Fourier transform to an accuracy of about 1%, experience has shown that it is necessary to execute the integration in Equation (2) for four orders of magnitude of time either side of the critical time constant. So, for instance, if the correlation function G(t) is determined by the motion of water at the pore surface, with the diffusion correlation time of about 1 μs, G(t) must be calculated for *t* from sub-nanosecond to milliseconds. Executing the Fourier transform over eight orders of magnitude of time takes a very substantial amount of computing time if standard Fourier transform packages are used. We have found it necessary to produce a bespoke Fourier transformation code which computes Equation (2) time decade by time decade. If the Fourier integrations are undertaken for *three* orders of magnitude of time either side of the critical time constant, the error is about 10%.

The relaxation rates $R_{1}\left(f\right)$ and $R_{2}\left(f\right)$ are now easily found from [38]:

(3) $$\begin{array}{ccc} R_{1}\left(\omega \right)= & = & \frac{1}{3}\beta_{IS}\left[7J\left(\omega_{S}\right)+3J\left(\omega \right)\right] \end{array}$$

(4) $$\begin{array}{ccc} R_{2}\left(\omega \right)= & = & \frac{1}{6}\beta_{IS}\left[4J\left(0\right)+13J\left(\omega_{S}\right)+3J\left(\omega \right)\right] \end{array}$$

for systems with paramagnetic impurities, or

(5) $$\begin{array}{ccc} R_{1}\left(\omega \right)= & = & \frac{1}{5}\beta_{II}\left[J(\omega )+4J(2\omega )\right] \end{array}$$

(6) $$\begin{array}{ccc} R_{2}\left(\omega \right)= & = & \frac{1}{10}\beta_{II}\left[3J(0)+5J(\omega )+2J(2\omega )\right] \end{array}$$

if paramagnetics are absent, where ω=2πf. Here, $\beta_{IS}={\left(\mu_{0}/4\pi \right)}^{2}\gamma_{I}^{2}\gamma_{S}^{2}\;\hbar^{2}S\left(S+1\right)$, $\gamma_{S}$ ($\gamma_{I}$) is the gyromagnetic ratio for the paramagnetic impurity (proton) with $S\;=\;\frac{5}{2}$ for the common paramagnetic impurities Fe${}^{3+}$ and Mn${}^{2+}$. The Larmor angular frequency of the impurity spin in the applied static field is $\omega_{S}\;=\;658.21\omega$. In Equation (5), $\beta_{II}\;=\;{\left(\mu_{0}/4\pi \right)}^{2}\gamma_{I}^{4}\;\hbar^{2}I\left(I+1\right)$, $\gamma_{I}$ is the proton gyromagnetic ratio and $I\;=\;\frac{1}{2}$. For water, $\beta_{II}\;=\;4.275\times 10^{5}$ nm${}^{6}$ s${}^{-2}$. Expressions for both $R_{1}$ and $R_{2}$ are presented because of the value of calculating the ratio $T_{1}/T_{2}=R_{2}/R_{1}$ at a spot frequency.

### 2.3. Comparison of Models

Porous materials are complex mixtures of solids, liquids and gases. The interpretation of FFC-NMR relaxation rate dispersion curves is extremely challenging, more so because of the complexity and diversity of the fluid environments. Nonetheless, a variety of simplified models have elucidated the key physics and provided interpretations of experimental data. Especially notable are the works of Korb, Kimmich, Levitz and co-workers [3,4,5,6,7,24,25,39,40,41,42,43,44] over the past three decades. These models fall into two broad categories: the Korb models, and a collection of models that we label as bulk-mediated surface diffusion (BMSD) models. If the FFC-NMR technique is to produce reliable characterising material parameters for porous materials, the underpinning physics and range of applicability of all model classes must be understood.

The Korb models [3,4,5,6,7,39,40] are applicable to systems in which the relaxation is dominated by the presence of paramagnetic impurities. Relaxation is fastest when the electronic spins of the paramagnetic ions are closest to the mobile proton spins because the interaction is stronger. Korb assumed that the surface water interacted with an effective layer of paramagnetic impurity density at the pore surface with both spin species lying in the same two-dimensional (2D) plane (1D models also exist). If spin-pair vectors lie in the same plane, the dipolar correlation function $G\left(t\right)\propto t^{-1}$ for long times. This relationship is exploited to construct an approximate expression for G(t) in terms of the time constants $\tau_{\ell }$ and $\tau_{d}$ (in our notation) which can be Fourier transformed analytically to yield a closed-form expression for $R_{1}\left(f\right)$.

The primary shortcoming of the Korb models is that the value of $\tau_{\ell }$ emerging from fits to FFC-NMR dispersion curves is several orders of magnitude smaller than $\tau_{d}$. This implies that water molecules make 100–10,000 or more hops on the surface before desorption, in disagreement with molecular dynamics simulations and other models [33,34,35]. The source of the difference in time scales is the construction of the dipolar correlation function G(t), which disallows a similarity of $\tau_{\ell }$ and $\tau_{d}$. The two time constants are, by necessity, different. The consequence is that fits to experimental dispersion curves reliably yield $\tau_{d}$ only. Nonetheless, the model is simple, quick and easy to use and $\tau_{d}$ is a useful physical quantity to extract from FFC-NMR measurements.

The second major class of model used to fit to FFC-NMR relaxation rate measurements assumes a non-Fickian surface diffusion of water at pore surfaces. The picture is of water molecules making Fickian diffusive jumps at the pore surface before desorption, followed by rapid diffusion in the bulk water before returning to the surface. The outcome is the non-Fickian, or Lévy, surface diffusion of spins [45,46], which is characterised by a Lévy parameter α lying in the range 0 to 2. Special cases are α=2, in which normal Fickian diffusion is restored; and α=1, which describes Cauchy dynamics. For simplicity, we label models that invoke non-Fickian surface dynamics collectively as BMSD models.

**Figure 3:**
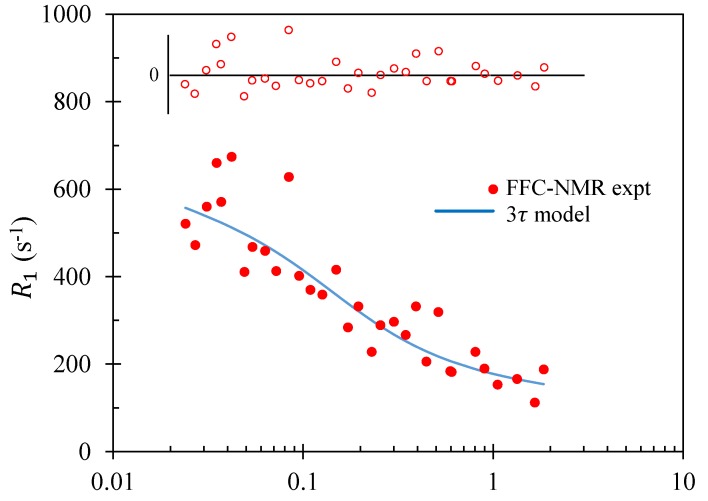
Fast-field-cycling nuclear magnetic resonance (FFC-NMR) spin–lattice relaxation rate data for a hydrated mortar by Barberon et al. [10], presented as a function of frequency. The solid curve represents the best fit obtained using the 3τ model and the 3TM fitting package. The open circles represent the residuals with the horizontal line indicating a residual of zero.

**Figure 4:**
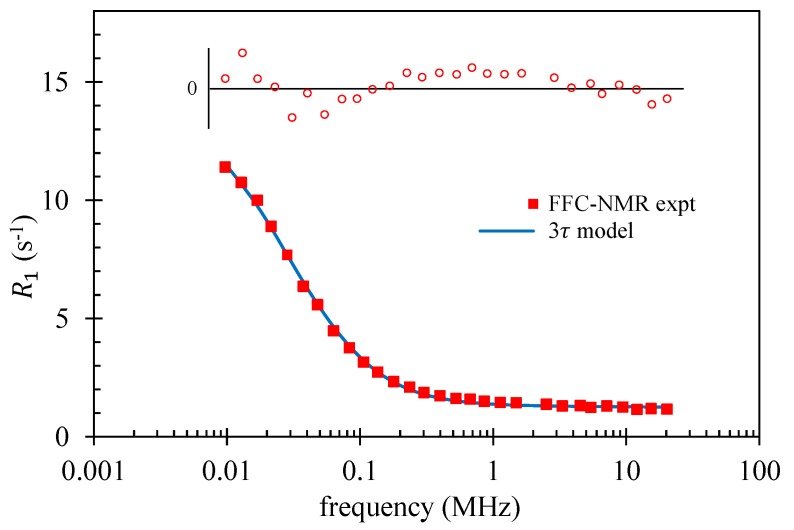
FFC-NMR spin–lattice relaxation rate data for a plaster paste by Korb [5], presented as a function of frequency. The solid curve represents the best fit obtained using the 3τ model and the 3TM fitting package. The open circles represent the residuals with the horizontal line indicating a residual of zero.

The motivation for introducing complex surface dynamics is the observed frequency dependence of the relaxation rate decay that emerges from many FFC-NMR experiments, as seen in Figure 3 and Figure 4. The form of $R_{1}\left(f\right)$ is associated with the functional form of G(t) and the long-time limit of G(t) is dictated by α. The outcome is that a fit to the decaying portion of FFC-NMR dispersion curves using a BMSD model yields a single time constant $\tau_{\ell }$. Its value is in agreement with $\tau_{\ell }$ from 3τ [31] and with $\tau_{d}$ from the Korb model. Moreover, BMSD models have been applied to systems in which relaxation is *not* associated with paramagnetic ions.

A shortcoming of the BMSD models is that analysis is normally restricted to the case α=1, or Cauchy dynamics, because a simple expression for G(t) is available. Furthermore, the justification for invoking Lévy dynamics is weak in porous materials with solid surfaces because water that has desorbed from a surface into the bulk is rapidly transported away from the desorption site to rejoin the surface some distance away where its contribution to relaxation is negligible. The long-time behaviour of G(t), which is determined by the Lévy parameter α, only becomes apparent in the low-frequency region of the $R_{1}\left(f\right)$ dispersion curve at frequencies below those accessible in the experiment. Consequently, Lévy dynamics may not influence the dispersion curves in systems with solid pore surfaces but may be important in soft porous systems where water may be retained in the surface environment and only briefly transits through the fast-moving environment.

A summary of physical quantities that may be obtained from an FFC-NMR measurement using each of the Korb, BMSD and 3τ models is supplied in Table 1. It is clear that the 3τ model is effective at maximising the outputs from an FFC-NMR measurement. However, 3τ lacks a neat, simple-to-use analytical expression for $R_{1}\left(f\right)$ that can be fit to experiment. Computations are required to obtain G(t) from Equation (1) and the Fourier transformation of Equation (2) must be undertaken numerically.

**Figure 5:**
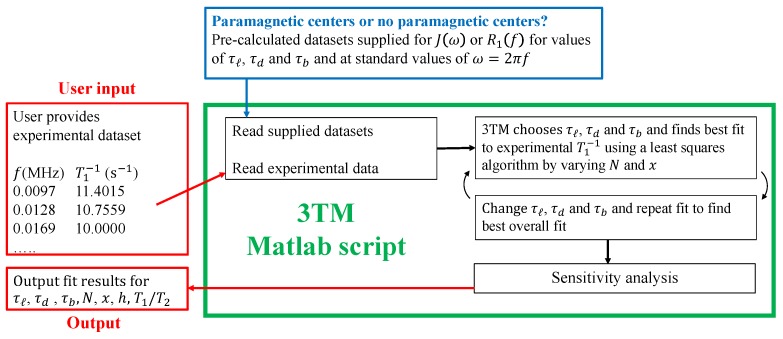

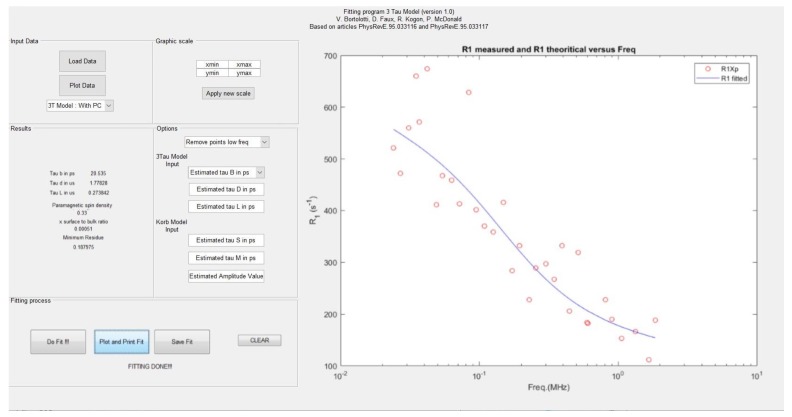
(**top**) The flow diagram provides a simplistic illustration of the function of the 3TM fitting package used to interpret FFC-NMR dispersion data. (**bottom**) A screen grab of the interface post-fitting is presented.

For this reason, we produced a software package in the form of a MATLAB code called 3TM, which allows users to execute a fit to FFC-NMR dispersion curves for a very wide range of porous materials. The relaxation rates have been pre-calculated for a set of time constants $\tau_{\ell }$, $\tau_{d}$ and $\tau_{b}$. 3TM reads in the pre-calculated data sets, the experimental data, and executes a fit using a choice of least squares approaches. A sensitivity analysis is undertaken that presents the ranges of values of the fit parameters within a user-specified criterion. A flow diagram and an example interface screen are provided as Figure 5. Researchers who have been unable to interpret their data using alternative models, or who wish to maximise the amount of information that can be gleaned from their experiments, may be able to extract the information listed in Table 1 using 3TM by contacting one of the authors. To illustrate its use, 3TM was used to interpret some historic FFC-NMR data in Section 3.

## 3. Results

The fitting package 3TM allows the 3τ model to be fit to FFC-NMR experimental dispersion curves obtained from systems with or without paramagnetic relaxation centres. 3TM is a MATLAB script with an interface that allows the user to read in their experimental FFC-NMR data and select fitting options. The best fit is obtained by minimising a conventional or logarithmic weighted least-squares fit parameter. The fitting process is summarised in Figure 5.

To illustrate the application of 3TM, two sets of historic experimental data were re-analysed: (i) a hydrated mortar in which relaxation is dominated by the presence of paramagnetic Fe${}^{3+}$; and (ii) a plaster paste sample in which there were no detectable paramagnetic ions. The optimum fit was determined by minimising an un-weighted $\chi^{2}$ least squares parameter. The fit parameters were $\tau_{\ell }$, $\tau_{d}$, $\tau_{b}$, *N*, and *x* where *N* is the paramagnetic impurity density, or the surface water density for the mortar or plaster respectively, and *x* is the planar pore surface-to-volume ratio.

### 3.1. Mortar

Historic FFC-NMR spin–lattice relaxation rate data for a hydrated mortar by Barberon et al. [10] are presented as a function of frequency in Figure 3. The solid curve represents the best fit obtained using 3TM, with summary of the results presented in Table 2. The scatter in the experimental data ensures that a range of values of the fit parameters yield similar values of $\chi^{2}$. To gauge the spread of “good” values of the fit parameters, a set of fits were extracted which differed from the optimum $\chi^{2}$ by no more than 1%. The outcome of these results are also presented in the table as a spread of values.

All values of the fit parameters obtained are physically reasonable. The bulk diffusion time constant for water at room temperature is 5.3 ps and the value obtained here, 21 ps is in excellent agreement. Diffusion of the bulk water in the mortar sample would be slower than pure water due to the presence of dissolved ions that bind to water and hinder diffusion, and because the slower-moving second hydration layer of water at the surface which constitutes the first “layer" of bulk water will dominate the relaxation.

The optimum fit was achieved with $\tau_{\ell }=0.27\;\mu$s and $\tau_{d}=1.8\;\mu$s. Note the 4–5 orders of magnitude difference in the time scales between these values and $\tau_{b}$. The number of hops that a molecule of water makes at the surface of the pore before desorption obtained from $\tau_{d}/\tau_{\ell }$ was quite large, at 6.5. This is indicative of relatively weak binding of the water to the surface and is a conclusion supported by the short value of $\tau_{\ell }$. It is also a conclusion supported by some unpublished molecular dynamics simulations [47] which indicate that in an idealised Lennard-Jones system, the ratio $\tau_{d}/\tau_{\ell }$ decreases as the binding energy between the water and the surface increases. However, the water surface *density* is also a factor in determining the ratio $\tau_{d}/\tau_{\ell }$ since a water molecule which is vibrating in its potential well at surface site will be less likely to find a neighbouring vacant site to hop to if the neighbouring sites are more distant. Therefore, this result is only suggestive of a low surface affinity of the water to the mortar.

It was not expected that the optimum fit result for the effective paramagnetic ion density would agree with independent experimental measurement, yet the value of 0.028 spins/nm${}^{3}$ is remarkably close to the result of 0.03 spins/nm${}^{3}$ obtained by electron spin resonance techniques in this case [10]. The characteristic pore dimension, at 0.74 μm, is also a physically reasonable result. This was calculated from the surface-to-volume ratio, *x*, on the assumption of planar pores and two surface layers each of thickness 0.27 nm in accordance with the 3τ model.

The scatter of the experimental data ensures a shallow $\chi^{2}$ surface in parameter space. An indication of the spread of values was obtained by considering all fits in which $\chi^{2}$ was within 1% of its optimum value. The range of values obtained is presented in Table 2. Here the relaxation time ratio $T_{1}/T_{2}$ at 20 MHz is presented. The ratio may be obtained experimentally from separate NMR $T_{1}$–$T_{2}$ correlation measurements at a fixed frequency. This ratio is not available for the mortar in [10], but a cement paste sample yields a value of about 4 at 20 MHz [15], in acceptable agreement with the values from 3TM.

### 3.2. Plaster

In this example, the 3τ model was used to analyse a plaster paste sample published in 2011 [5]. Tests indicate no paramagnetic impurities were present. This set of experimental data was not subjected to a fit in the original paper. The results from the fits using 3TM are summarised in Table 3 and plotted in Figure 4.

The values of the time constants $\tau_{\ell }$ and $\tau_{d}$ were longer than those for the mortar and the ratio $\tau_{d}/\tau_{\ell }$ was 2.7—both indicative of stronger water–surface interactions. The quality of the FFC-NMR data allows the opportunity to treat $N_{\ell }$, the surface water spin density, as a fit parameter. The best fit produced $N_{\ell }=73$ spins/nm${}^{3}$ which is reassuringly close to the bulk water value of 66.6 spins/nm${}^{3}$.

The planar-pore-equivalent pore thickness was 0.38 μm—approximately half that of the mortar. Finally, and impressively, the ratio $T_{1}/T_{2}$ was found to be 3.9. 3TM fits three time constants to $R_{1}\left(f\right)$, where each time constant can, in principle, span picoseconds to microseconds. The range of values of $\tau_{\ell }$, $\tau_{d}$ and $\tau_{b}$ that secured a high-quality fit was extremely narrow. The quality of the experimental data was so good that the $\chi^{2}$ surface had a very sharp minimum in parameter space. The ratio $T_{1}/T_{2}$ obtained from separate NMR $T_{1}$–$T_{2}$ correlation measurements from a cement paste, a similar material, is about 4. Calculations using 3TM found $T_{1}/T_{2}\approx 4$ only in a very narrow range of $\tau_{\ell }$, $\tau_{d}$ and $\tau_{b}$. The coincidence of the two sharp minima in the $(\tau_{\ell }$, $\tau_{d}$, $\tau_{b})$ and $T_{1}/T_{2}$ spaces provides compelling evidence of the veracity of the 3τ model.

## 4. Discussion and Conclusions

The 3τ model was presented in 2017 as a means of interpreting FFC-NMR dispersion curves obtained from porous materials [30,31]. However, it was difficult to fit the model to experimental data in practice due to the complexity of the calculations required to obtain $R_{1}$. In this article, a MATLAB script code called 3TM is introduced which removes this difficulty. The code reads in pre-calculated data sets based on the 3τ model and executes a fit by minimising a least squares parameter. The results presented here validate 3TM for a mortar and a plaster paste yielding physically reasonable fit parameters in both cases. The 3τ model was shown to extract a large number of physical parameters from $R_{1}\left(f\right)$ relaxation rate dispersion curves, whether relaxation was dominated by the presence of paramagnetic relaxation centres or not.

The 3τ model constitutes the most effective model for the interpretation of FFC-NMR dispersion data from porous materials with solid surfaces, but the model is un-tested for soft porous materials such as hydrogels, polymer systems and biological material. We are doubtful that the 3τ model would prove useful for soft porous material without adaptation, and this remains a target for future work. Alternative models to 3τ, such as the Korb and BMSD models, remain useful because they are quick and easy to apply. It is important to be cognisant of the limitations of all models. The Korb model is incorporated into our fitting package, as seen in Figure 5, and can be used as a comparator for the desorption time parameter $\tau_{d}$ which is reliably obtained.

A version of the 3TM package will be made available for free download through GitHub [48].

## Figures and Tables

**Table 1:** List of physical quantities that may be provided by an FFC-NMR measurement from a porous material by the Korb, bulk-mediated surface diffusion (BMSD) and 3τ model types.

Quantity	Korb	BMSD	3τ	Comments
$\tau_{\ell }$	✓	✓	✓	Diffusion correlation time for fluid at the pore surface.
$\tau_{d}$	✓		✓	Desorption time constant for the surface fluid.
$\tau_{b}$			✓	Bulk fluid diffusion time constant related to the diffusion coefficient.
$N_{\sigma }$			✓	Each model employs a scaling factor proportional to the number of paramagnetic ions per unit volume in the solid. 3τ provides the equivalent spin density for the effective paramagnetic layer.
$N_{\ell }$			✓	For water, the surface spin density is normally set to 66.6 spins/nm${}^{3}$ as for the bulk. $N_{\ell }$ can act as an additional fit parameter within the 3τ model.
x≈S/V			✓	The dimensionless ratio of the volume of the pore surface (thickness assumed to be δ= 0.27 nm) to the pore volume.
*h*			✓	The “planar-pore-equivalent" pore thickness is equal to $5.4\times 10^{-4}/x$ in units of μm. Useful characteristic pore dimension.
α		✓	✓	The Lévy parameter is a measure of the departure from Fickian dynamics. α=1 in most BMSD models. Lévy dynamics is trivially introduced into 3τ but is not necessary to secure good fits to FFC-NMR data.
$\tau_{d}/\tau_{\ell }$	✓		✓	The ratio of time constants is approximately equal to the number of hops a spin makes at a surface before desorption. It is linked to surface affinity (see text).
$T_{1}/T_{2}$	✓	✓	✓	The ratio of spin–lattice relaxation time to the spin–spin relaxation time is sometimes available at a spot frequency from separate $T_{1}$–$T_{2}$ correlation measurements. Easily estimated by any model for comparison.

**Table 2:** Results of a fit of the 3τ model to a FFC-NMR dispersion using 3TM for a hydrated mortar due to Barberon and co-workers [10].

Quantity	Value	Comments
$\tau_{\ell }$	0.27 μs	Best fit result for the surface water diffusion correlation time.
$\tau_{d}$	1.8 μs	Best fit desorption time constant.
$\tau_{b}$	21 ps	Best fit bulk water diffusion time constant equivalent to a diffusion coefficient of 0.6 × 10${}^{-9}$ m${}^{2}$s${}^{-1}$
$N_{\sigma }$	0.028 ions/nm${}^{3}$	The best fit paramagnetic ion number density is close to the measured value of 0.03 [10].
*x*	0.00073	The best fit surface-to-volume ratio.
*h*	0.74 μm	The planar-pore-equivalent pore thickness.
$\tau_{d}/\tau_{\ell }$	6.5	The number of surface hops of water before desorption.
$\tau_{\ell }$	0.15–0.42 μs	Range of values obtained from good fits (see text).
$\tau_{d}$	1.2–2.1 μs	Range of values obtained from good fits (see text).
$\tau_{b}$	21 ps	All good fits yielded the same value of the bulk diffusion time constant.
$<\;\tau_{\ell }\;>$	0.24 μs	Mean value of $\tau_{\ell }$ from the spread of good fits.
$<\;\tau_{d}\;>$	1.5 μs	Mean value of $\tau_{d}$ from the spread of good fits.
$T_{1}/T_{2}$	2.9–3.3	Range of values obtained from the set of good fits. The experimental value is typically 4 for cement paste at f= 20 MHz [15].

**Table 3:** Results of a fit of the 3τ model to an FFC-NMR dispersion for a plaster paste from Korb [5] using 3TM.

Quantity	Value	Comments
$\tau_{\ell }$	2.4 μs	Best fit result for the surface water diffusion correlation time.
$\tau_{d}$	6.5 μs	Best fit desorption time constant.
$\tau_{b}$	13 ps	Best fit bulk water diffusion time constant equivalent to a diffusion coefficient of 0.9 × 10${}^{-9}$ m${}^{2}$s${}^{-1}$
$N_{\ell }$	73 spins/nm${}^{3}$	The best fit ${}^{1}$H spin density for the surface layer is similar to the 66.6 spins/nm${}^{3}$ for bulk water.
*x*	0.00144	The best fit surface-to-volume ratio.
*h*	0.38 μm	The planar-pore-equivalent pore thickness.
$\tau_{d}/\tau_{\ell }$	2.7	The number of surface hops of water before desorption.
$T_{1}/T_{2}$	3.9	The experimental value is typically 4 f= 20 MHz for cement paste [15].

## Abbreviations

The following abbreviations are used in this manuscript:

NMR	nuclear magnetic resonance
FFC-NMR	fast-field-cycling NMR
BMSD	bulk-mediated surface diffusion
